# A multi-objective optimisation approach with improved pareto-optimal solutions to enhance economic and environmental dispatch in power systems

**DOI:** 10.1038/s41598-024-62904-4

**Published:** 2024-06-11

**Authors:** Muhammad Ilyas Khan Khalil, Izaz Ur Rahman, Muhammad Zakarya, Ashraf Zia, Ayaz Ali Khan, Mohammad Reza Chalak Qazani, Mahmood Al-Bahri, Muhammad Haleem

**Affiliations:** 1 Department of Computer Science, Abdul Wali Khan University, Mardan, Pakistan; 2 Faculty of Computing and Information Technology, Sohar University, Sohar, Oman; 3 Department of Computer Science, University of Lakki Marwat, Lakki Marwat, Pakistan; 4 Department of Computer Science, Kardan University, Kabul, Afghanistan

**Keywords:** Particle swarm optimisation, Markov chain, Evolutionary factor, Large-scale optimisation, Scalability, Power distribution, Computer science

## Abstract

This work implements the recently developed *n*th state Markovian jumping particle swarm optimisation (PSO) algorithm with local search (NS-MJPSO*loc*) awareness method to address the economic/environmental dispatch (EED) problem. The proposed approach, known as the Non-dominated Sorting Multi-objective PSO with Local Best (NS-MJPSO*loc*), aims to enhance the performance of the PSO algorithm in multi-objective optimisation problems. This is achieved by redefining the concept of best local candidates within the search space of multi-objective optimisation. The NS-MJPSO*loc* algorithm uses an evolutionary factor-based mechanism to identify the optimum compromise solution, a Markov chain state jumping technique to control the Pareto-optimal set size, and a neighbourhood’s topology (such as a ring or a star) to determine its size. Economic dispatch refers to the systematic allocation of available power resources in order to fulfill all relevant limitations and effectively meet the demand for electricity at the lowest possible operating cost. As a result of heightened public consciousness regarding environmental pollution and the implementation of clean air amendments, nations worldwide have compelled utilities to adapt their operational practises in order to comply with environmental regulations. The (NS-MJPSO*loc*) approach has been utilised for resolving the EED problem, including cost and emission objectives that are not commensurable. The findings illustrate the efficacy of the suggested (NS-MJPSO*loc*) approach in producing a collection of Pareto-optimal solutions that are evenly dispersed within a single iteration. The comparison of several approaches reveals the higher performance of the suggested (NS-MJPSO*loc*) in terms of the diversity of the Pareto-optimal solutions achieved. In addition, a measure of solution quality based on Pareto optimality has been incorporated. The findings validate the effectiveness of the proposed (NS-MJPSO*loc*) approach in addressing the multi-objective EED issue and generating a trade-off solution that is both optimal and of high quality. We observed that our approach can reduce $\sim$6.4% of fuel costs and $\sim$9.1% of computational time in comparison to the classical PSO technique. Furthermore, our method can reduce $\sim$9.4% of the emissions measured in tons per hour as compared to the PSO approach.

## Introduction

In the power industry, recent research has been driven to focus on issues related to reviewing industrial design or operations in order to lower pollution and emissions to the environment. These ecological effects usually appear from thermal power stations^1–3^ due to the growing public awareness of environmental protection. This is due to the fact that these power stations consume significant amount of fuel and, most recently, the world has seen rising fuel costs along with its environmental impacts. The emission dispatching option, which aims to reduce both emissions and fuel costs, is a desirable short-term alternative. This strategy has attracted a lot of interest recently^4–8^ because it only needs a slight modification to the fundamental economic dispatch to account for emissions.

In power systems, one of the most important optimisation problems is known as Economic Environmental Dispatch (EED) that is sometimes referred to as Economic Emission Dispatch (EED). The EED’s primary objective is to ascertain which configurations of components within a power generation system result in the most efficient generation of power. The catch is, however, that the solution needs to be both economically viable and environmentally friendly in order to be considered acceptable. The significance of the EED problem has been growing substantially as people all over the world become more aware of the need to preserve the natural environment. The goal is crystal clear: to simultaneously cut down on the overall expense of fuel and the pollution that it causes to our environment. The PSO has become an increasingly popular method for addressing the EED problem over the course of the past several years. This is mostly attributable to the fact that it is a straightforward method that is both effective and good at locating global optimal solutions to similar optimisation problems.

The ELD problem of power systems has been successfully resolved by using PSO techniques as discussed in section Related work. In the existing literature, PSO algorithms iteratively modify the parameters of a swarm of particles to converge toward the ideal solution by maximizing the distribution of power generation across numerous units while taking into account several restrictions including fuel cost, power demand, and generator limits. To meet the real-time requirements for dynamic power system operation, however, more developments are still required to improve the performance of PSO methods for ELD, particularly in handling larger and more complex power systems, integrating renewable energy sources, taking uncertainties into account, and increasing computational efficiency. Furthermore, we believe that there still exists a gap to concentrate on creating advanced PSO-based strategies that combine PSO’s advantages with other optimisation methods to get around traditional PSO algorithms’ drawbacks and produce more durable and dependable solutions for the ELD issue in contemporary power systems.

This paper proposes and employs a novel *n*th state Markovian jumping PSO algorithm with a local search (NS-MJPSO*loc*) method to solve the economic/environmental dispatch problem. Subsequently, the newly developed algorithm implies the theory of local search capability. Using this capability, the problem search space having multiple optima is thoroughly explored. It is known that the canonical versions of the PSO algorithms are based on global search. Similarly to other multi-objective evolutionary algorithms, an evolutionary factor-based mechanism is used to identify the optimum compromise solution, and a Markov chain state jumping technique is used to control the Pareto-optimal set size. The results of several runs on the common IEEE topology test system are compared to other methods described in the literature. The efficacy and potential of the proposed (NS-MJPSO*loc*) approach are shown to solve the multi-objective EED problem. The major contributions of this research are as follows:

- we propose and employ a novel *n*th state Markovian jumping PSO algorithm with local search (NS-MJPSO*loc*) method to solve the economic/environmental dispatch problem;
- the proposed algorithm uses an evolutionary factor-based mechanism to identify the optimum compromise solution;
- a Markov chain state jumping technique is used to control the Pareto-optimal set size along with a neighbourhood’s topology (such as a ring or a star) to determine its size; and
- the algorithm is implemented for the economic dispatch problem in the domain of power systems. The experimental outcomes of the proposed NS-MJPSO*loc* approach has been verified on IEEE 30 Bus and 15-unit Systems.

The rest of the paper is organized as follows. A summary of the related work is offered in Section Related work. The problem statement along with the constraints is explained in section Problem statements. The optimisation problem is formulated in section Problem formulation. The concept of the multi-objective optimisation is elaborated in section The concept of multi-objective optimisation. The proposed multi-objective optimisation algorithm is explained in section The proposed NS-MJPSOloc algorithm. Performance evaluation of the proposed algorithm is discussed in section Performance evaluation. The experimental setup is explained in section Experimental setup and evaluation metrics are given in section Evaluation metrics. The obtained results and findings are illustrated in section Results and discussion. Finally, the concluding remarks along with future research directions are summarized in section Conclusions and future work.

### Nomenclature

The list of abbreviations shown in Table 1 and the list of mathematical notations shown in Table 3 are used in the rest of the paper. We believe that these tables will help all readers to quickly understand all the mathematical formulas mentioned in this paper.

**Table 1:** List of Abbreviations.

Abbreviation	Description
PSO	Particle swarm optimisation
ELD	Economic load dispatch
NS-MJPSO_loc_	N-states Markovian jumping particle swarm optimisation with local-best
EED	Economic and Emission Dispatch
CPPS	Cyber-Physical Power System
FIDA	False Data Injections Attack
CMOPEO-EED	Constrained multi-objective population extremal optimisation based economic-emission dispatch
PCPSO	Perfectly convergent particle swarm optimisation
MOP	Multiobjective optimisation problem
MOEA	Multiobjective optimisation evolutionary algorithm
MaOP	Many objective optimisation problem
MPSO	Modified particle swarm optimisation
VEPSO	Vector evaluated particle swarm optimisation
DEED	Dynamic economic environmental dispatch
CSA	Cuckoo search algorithm
HFA	Hybrid firefly algorithm
ACO	Ant colony optimisation
GA	Genetic algorithm
EP	Evolutionary programming
ES	Evolutionary strategies
DE	Differential evolution
SA	Simulated annealing
HCA	Hill climbing algorithm
NS-MJPSO	N-states Markovian jumping particle swarm optimisation
NS-SPSO	N-states switching particle swarm optimisation
CEED	Combined economic emission dispatch

## Related work

In 2014 Han et al.^9^ delved deep into the environmental and economic dispatch of a micro-grid, encompassing diverse energy sources like photovoltaic generation, wind turbines, and more. The study underscored the efficacy of an improved linearly decreasing weight PSO algorithm, emphasizing its theoretical and practical feasibility. In 2016 Tlijani et al.^10^ presented an extended version of the conventional DEED (dynamic economic environmental dispatch), aiming to mitigate ramp rate violations across consecutive dispatch periods. The goal was clear: to consistently meet periodic load demands. In 2017^11^ illuminated the potential of a multi-objective PSO algorithm, leveraging both the Pareto criterion and fuzzy logic, to address environmental pollution in economic dispatch. In 2018^12^ elegantly formulated the power dispatch challenge as a dual-objective optimisation problem. The mission was dual-pronged: simultaneous minimization of fuel cost and emissions.

Considering its concealed nature, false data injection attacks (FDIAs) have attracted a lot of attention in the field of cyber-physical power systems (CPPS). Improving CPPS cybersecurity requires an understanding of likely attacker actions. Nonetheless, the majority of FDIA models now in use frequently concentrate on the implications of attacks or the effects of attackers alone. In response, a unique multi-objective stealthy FDIA strategy is presented in^13^ within the framework of an AC grid model. In order to maximize the impact of the attack and minimize tainted measurements while keeping stealth, the suggested attack model is presented as a multi-objective optimisation problem. Additionally, in order to improve the attack vector’s generation efficiency, a novel representation mechanism is proposed to characterize the positions and parameters of injected states^13^.

In 2019^14^ proposed a refined version of PSO to tackle the EED conundrum of thermal electric power units. The innovative Space Reduction strategy was employed to pinpoint the Pareto optimal solution within the designated search space. In 2020^15^ integrated DE with Quantum PSO (QPSO) to address the short-term EED challenge of microgrids. In another study, Mehrpour et al.^16^ focused on the dynamic load and emission dispatch in daily cycles, especially considering the potential impacts of renewable energy sources. In 2022^17^ showcased the Perfectly Convergent PSO (PCPSO) for addressing combined economic and multiple emissions dispatch challenges. The study meticulously considered the ramifications of various pollutants, employing cubic functions with seven price penalty factors. The history of EED when viewed through the perspective of these research articles, presents a picture of continuous innovation and development. Microgrids and other forms of renewable energy are only two examples of how this industry has expanded its scope in pursuit of solutions that are good for the economy while also being friendly to the environment. The application and refining of PSO have been the constant thread spinning throughout this exploration, establishing its strength as a solid tool for EED difficulties.

Environmental/economic dispatch (EED) problems have been resolved in a variety of ways^4–8,18–22^. In general, there are three methods to resolve the EED issue. The first approach involves considering the level of emissions as a constraint with a tolerable limit^4^. However, demonstrating the relationship between cost and emissions in this formulation is quite difficult.

The second method addresses the emission as a separate objective beside the traditional cost objective^5–8^. However, the EED problem was simplified to a single objective function by linearly combining the two objectives or only taking into account a single objective at each stage of optimisation. inevitably, this method finds marginally non-dominated solutions and necessitates more runs than the required number of Pareto-optimal solutions. In power systems, the Economic Emission Dispatch (EED) problem is a well-known constrained multi-objective optimisation problem. It strives to meet a variety of operational requirements while concurrently minimizing expenses and emissions. Even though a lot of solutions have been devised to deal with it, however, this problem remains difficult and challenging because of the unpredictable and inconsistent nature of renewable energy sources (RES) like wind and solar, in particular, when they are integrated into the system. The authors in^23^ present a novel constrained multi-objective optimisation technique called CMOPEO-EED, with the goal of improving EED performance in the presence of renewable power generation.

The third method treats fuel cost and emission as dual objectives at the same time. For the EED issue, multi-objective search heuristic and fuzzy membership-based optimisation approaches have been explored^18,19^, and^20^, whereas, algorithmic approaches do not offer a logical framework for guiding the search to the Pareto-optimal front, and it is extremely hard to expand these methods to include other objectives. These methods need a lot of computational power and, subsequently, take a lot of time. Numerous non-dominated alternatives can be observed in a single run using strategies based on multi-objective genetic algorithms, as described in^21,22^. Premature convergence is a problem with genetic algorithm-based methods, and the method described in^21^ requires a lot of computational work because of the ranking process that takes place throughout the fitness assignment phase.

The PSO approach provides an adaptable and diverse strategy to enhance and evolve global and local exploration capabilities, despite genetic algorithms and heuristic approaches. Compared to the genetic algorithm, it typically produces faster convergence rates^24^. PSO has been applied with remarkable popularity in the past decade to a variety of power system problems, such as the economic power dispatch issue^25,26^, and^27^. It has been shown and documented that PSO has the potential to deal with non-smooth and non-convex economic power dispatch problems^26,27^. subsequently, the fuel cost was the only factor taken into account for optimisation when the problem was defined as an ordinary dispatch problem.

In order to obtain an edge of optimal solutions, it is predominantly necessary to redefine global and local best persons when switching from a uni-objective to a multi-objective PSO. There is a set of non-dominated solutions rather than an absolute global best in multi-objective PSO. Additionally, there could not be a single local best individual for every swarm particle. In a multi-objective domain, selecting the global and local best for steering the swarm particles turns into a challenging problem.

Numerous real-world problems can be formulated as multi-objective optimisation problems (MOPs), in which it is necessary to simultaneously optimize several, frequently conflicting objectives. Finding a set of solutions that are unable to be enhanced in one area without compromising another is the aim of addressing a mixed optimal problem (MOP). MOEAs, or multi-objective evolutionary algorithms, have become a popular and successful method for handling MOPs^28^. As MOEAs may produce roughly optimum solutions in a single run and do not require specific assumptions like continuity or differentiability, they are used in the majority of the related works. Furthermore, these methods are based on randomised search algorithms that draw inspiration from Darwin’s theory of natural selection. Although MOEAs have obvious benefits, it is vital to remember that they require a large number of objective function evaluations, which could make them unfeasible for some applications requiring a lot of computational power.

The computational complexity of evaluating the objective functions and the flexibility of the input parameters are the two main determinants of the computational cost of MOEAs at each iteration^29,30^. Since many MOPs in the first instance are unable to be represented analytically, evaluations must be done through laborious simulations. Though there have been several attempts to reduce the MOEA execution time by utilizing the knowledge collected throughout the search process, these approaches usually lead to a consensus regarding the accuracy level of the final findings. Applications using high-dimensional spaces, including multi-objective programming (MOP) problems with four or more objectives or those with hundreds or thousands of decision variables, fall under the second instance. Large-scale MOPs and many-objective optimisation problems (MaOPs) are the terms used to describe these kinds of MOPs^31^. They considerably increase MOEAs’ runtime^32^. Furthermore, large population size is needed for some applications in order to improve accuracy or cover more ground in the search space^33–35^. While the majority of MOEAs function within an expected polynomial time for the size of their population, storage capacity limitations can provide difficulties^36,37^.

Recent reports indicate that PSO variants including NS-MJPSO*loc* implementation require only a small amount of work to resolve power system issues. In order to handle the EED problem with thermal dispatch and various renewable energy sources, Wang and Singh^38^ proposed a fuzzified MOPSO. The method offers a fuzzification process for choosing the world’s top person while considering the world’s top as an entire area rather than simply a single spot. On the other hand, each particle is maintained with a single local best solution. This will reduce search efficiency and is against the multi-objective optimisation principle. By breaking down the original optimisation problem into smaller problems, Kitamura et al.^39^ presented a modified MOPSO to optimize an energy management system. This method, however, faces serious limitations when there is a significant interaction between the constraints in several sub-problems. In their MOPSO-based solution to the congestion management issue, Hazra and Sinha^40^ demonstrated how to reduce both costs and congestion at the same time. The sigma method^41^ is used in this strategy to identify the ideal local particle guide. However, the use of the sigma values heightens the PSO’s already considerable selection pressure. In some situations, such as multifrontal difficulties, this may lead to early convergence.

In order to calculate the contributions of generators to the transmission system, a vector-evaluated PSO (VEPSO) was suggested and studied^42^. Depending on each objective independently, VEPSO selects portions of the future generation from the older generation. However, choosing people who excel in one area without considering the other areas raises the issue of eliminating those with average performance, who might be very helpful in finding compromise solutions^43^. In^44,45^, the authors have proposed solutions for enhancements in evolutionary algorithms and optimization techniques^44^ outlines a strategy for improving the effectiveness of SAEAs (Surrogate-assisted evolutionary algorithms) utilizing unevaluated solutions. A crucial element of MOEA, the offspring generator, has received little attention and lacks a design concept. In order to overcome this problem, regularity evolution (RE), an offspring generation model for MOEAs, is introduced in^45^. The authors in^46^ proposed an adaptive algorithm for control system optimizations to address the issue of selecting optimal starting values for connection weight parameters in MPIDNN (multivariable PID neural network). The authors suggested a constrained optimization problem and an adaptive population extremal optimization-based MPIDNN method called PEO-MPIDNN, which minimizes exponential time and system errors.

In^47,48^, the authors have proposed novel approaches in multi-objective optimization and algorithm efficiency within machine learning environments. For online sequential learning machines, in^47^ a multi-objective model selection approach is suggested to enhance the target output error, control quality, and channel equalization. To build channel equalization models and accomplish classification selection and equalization, the proposed method makes use of feedback compensation and adaptive equalization control. The authors in^48^ suggest ASDNSGA-II, an improved fast NSGA-II based on an adaptive crossover technique and unique congestion strategy. The proposed approach balances the convergence and variety of the decision and object spaces, hence improving the selection strategy.

^49^ discusses the application of federated learning (FL) and blockchain technology in IIoT. To lower energy usage and application latency, the study focuses on FL Aware Multi-Objective Modeling in Decentralized Microservices Assisted IIoT Systems. To optimize workload allocation and application delay, the Blockchain-Enabled FL Algorithm Framework (DLEBAF) is designed with three strategies: (i) deadline-efficient task sequencing and scheduling (DETS), (ii) latency-efficient task scheduling (LETS), and (iii) energy-efficient task scheduling (EETS). Table 2 shows the advantages and disadvantages of the existing works. We believe the data in Table 2 will help our reader to quickly identify what is missing in the current literature and what is further needed to improve the efficiency of EED problems in power systems.

**Table 2:** Advantages and disadvantages of existing works.

Ref	Methods used	Strengths	Weaknesses
^50^	Hybrid firefly algorithm (HFA) Genetic algorithm (GA)	Hybrid FA-GA algorithm for environmental economic dispatch Improved criteria for determining global and local optima	The research does not address the issue of scalability in relation to the algorithm’s performance when applied to larger power systems that encompass a greater number of thermal power plants. The provided analysis lacks a comprehensive examination of the algorithm’s computational efficiency, specifically in terms of the duration necessary for convergence to the best solution.
^51^	Cuckoo Search Algorithm (CSA)	Solving economic dispatch problem using Cuckoo Search Algorithm Testing the method on western Algerian electrical power system	The work lacks an investigation of the computational complexity of the CSA as well as any discussion on its convergence features. Furthermore, the paper fails to address the CSA’s ability to effectively manage uncertainties or fluctuations within the power system, a critical aspect for practical implementation.
^52^	Crow search algorithm (CSA) Eagle strategy (ES)	Hybridization of crow search algorithm with eagle strategy Proposed solution for economic dispatch problem (EDP).	The operational expenses associated with pumped storage systems are minimal. Simultaneous operation of pumping and producing processes is not feasible in pumped storage units.
^53^	Bald Eagle Search (BES) optimisation algorithm WOA, GA, PSO, and GSA optimisation methods	Introducing the Bald Eagle Search (BES) optimisation algorithm Comparing the BES algorithm with other optimisation methods	The BES algorithm has the potential for expansion to encompass more categories of renewable energy sources, such as hydropower or biomass, thereby offering a more holistic approach to optimising power systems. The algorithm’s effectiveness can be improved by incorporating supplementary restrictions and parameters, such as transmission losses, voltage stability, and ramp rate limits, hence enhancing its suitability for real-world power systems.
^54^	Harmony search algorithm Ant colony optimisation (ACO)	Proposed hybrid algorithm (HSA-ACO) for EED problems Comparative analysis with other optimisation techniques	The study solely evaluates the efficacy of the suggested algorithm on power systems with 3-unit and 6-unit configurations, perhaps limiting its applicability to large-scale power systems. The publication lacks a comprehensive analysis of other optimisation approaches, which hinders the evaluation of the algorithm’s efficacy in comparison to established methods.
^55^	Levy-based glowworm swarm optimisation (LGSO) GA, Grey wolf optimisation, whale optimisation algorithm (WOA), dragonfly algorithm (DA) and glowworm swarm optimisation (GSO)	Introducing a meta-heuristic algorithm for CEED Proposing a novel algorithm called Levy-based glowworm swarm optimisation (LGSO).	The potential of the LGSO algorithm can be further optimised by integrating supplementary elements, such as grid constraints, ramp rate limits, and system stability considerations. Comparative analyses can be undertaken to assess the efficacy of the LGSO algorithm in comparison to other cutting-edge optimisation algorithms when addressing CEED concerns.
^56^	Multi-objective squirrel search algorithm (MOSSA) Squirrel search algorithm based weighted sum approach (SSA-WSA) with price penalty factors, artificial bee colony (ABC), and exchange market algorithm (EMA)	Introducing multi-objective squirrel search algorithm (MOSSA) Achieving preferred trade-off solutions over other algorithms.	The research does not address the constraints or potential disadvantages associated with the MOSSA approach, such as its computing complexity or susceptibility to variations in problem size and complexity. Furthermore, the analysis conducted utilising the MOSSA technique lacks comprehensiveness in terms of evaluating the statistical significance and robustness of the acquired data.
^57^	Metaheuristic optimisation techniques: moth-flame optimisation, salp swarm algorithm, improved grey wolf optimizer, and multi-verse optimizer Weighted sum strategy using the analytic hierarchy process (AHP)	ETED model for IEEE 30-bus system with RESs Metaheuristic optimisation techniques for cost, losses, and emissions.	System restrictions encompass equality and inequality limits that involve banned operation zones (POZs). Metaheuristic optimisation approaches are utilised in order to identify the optimal solution for several factors such as generation cost, losses, and emissions.
^58^	Hybrid Multi-verse Optimizer (MVO) hybridized with sequential quadratic programming (SQP) is proposed for the solution of multi-constrained ED problem	The presented mechanism demonstrates improved convergence features, numerical proficiency, and robustness in solving the multi-constrained ED problem.	Comparative analyses can be undertaken to assess the efficacy of the HMVO approach in relation to other advanced optimisation techniques, including GAs, PSO, and DE. These evaluations aim to ascertain the competitiveness and superiority of the HMVO approach in addressing the Economic Dispatch problem.

## Problem statements

In order to solve the EED problem, the fuel cost and emission objective functions should be minimized while adhering to a number of equality and inequality constraints. In section Problem objectives, we describe the objectives of EED problem. In section Problem constraints, various constraints are explained. Finally, the EED problem is mathematically described in section Problem formulation. Table 3 describes a list of all mathematical notations and their brief description. We believe that this table will help all readers to quickly understand all the mathematical formulas.

**Table 3:** List of mathematical symbols and notations.

Notation	Description
*j*	Particle
$v_j$	Particle velocity
$x_j$	Particle position
*D*	Problem’s dimensionality
*w*	Scaling factor
$rand_1$, $rand_2$	Random numbers, uniformly distributed, between [0, 1]
$c_1$, $c_2$	Cognitive and social acceleration coefficients
$Pbest_j$	Best solution identified by particle *j*th
$Gbest_j$	Best solution for particle *j*th throughout the swarm
$P = (p_ij)JJ$	The probability transition matrix
$E_f$	Evolutionary factor
$\delta$	Auxiliary parameter
$\omega$	inertia weight
*N*	Number of states
*n*	Population size
*f*	Benchmark function for evaluation
$P_d(min)$	Minimum mean distance of all particles $P_{d}(i)$
$P_d(max)$	Maximum mean distance of all particles $P_{d}(i)$
*pbest*	Particle local best position
*gbest*	Particle global best position
*loc*	Local search mechanism
*S*	Evolutionary state
$P_G$	Generator Power
$P_D$	Consumed Power
*E*	Emission
$\theta$	Voltage phase angle
$P_{loss}$	Power loss

The problem in question is typically formulated as follows.

### Problem objectives

We consider two objectives for the EED problem in our optimisation problem i.e. fuel cost and carbon emissions. The mathematical foundations of both objectives are described in subsequent subsections.

#### Smooth cost minimization

The goal of the ELD problem is to generate electricity while satisfying all system constraints at the lowest possible cost per unit (fuel cost in US dollars). The cost of each generator is calculated using the quadratic function in the smooth or simplified ELD problems. The equality and inequality constraints are the fundamental limitations of the simplified cost functions. The total cost of fuel in US dollars per hour, *F*(*P*), is represented as:

1 $$\begin{aligned} \sum _{i=1}^{N}F_{i}({P_{G}}_{i}) = \sum _{i=1}^{m}a_{i}+b_{i}{P_{G}}_{i}+c_{i}{P_{G}}_{i}^2 \end{aligned}$$

The objective function is a minimisation problem and is given by:

2 $$\begin{aligned} min(C.F) = \sum _{i=1}^{N}F_{i}({P_{G}}_{i}) = \sum _{i=1}^{m}a_{i}+b_{i}{P_{G}}_{i}+c_{i}{P_{G}}_{i}^2 \end{aligned}$$

where C represents the total generation cost and $F_i$ represents the objective function of generator *i*. Furthermore, *N* is the total number of generators, $a_i$, $b_i$, and $c_i$ are the *i*th generator’s cost coefficients, and ${P_{G}}_{i}$ is the generator’s real power output of the *i*th generator. ${P_{G}}_{i}$, which is described as follows.

3 $$\begin{aligned} P_{G_{i}} = [P_{i1},P_{i2},P_{i3}, \cdots , P_{iD}], i = 1,2, \cdots , n \end{aligned}$$

The index of a particle is represented by *n*, while the number of units or generators is represented by *D*. For example, $P_{i1}$ denotes the power produced by unit 1. The dimension of the problem in this function is [Population *times* Number of systems units]. However, on the basis of instances, appropriate constraints are taken into account.

#### Carbon emission minimization

In order to develop a mathematical model for emission reduction in a power system, the challenges including power generation, emission factors, and various constraints are being considered. A generalized mathematical model for minimizing emissions is provided here:

**Decision variables:** In the emission reduction model, we define the following decision variables.

$P_{Gi}:$ Power generated by the *i*th generator.

$E_{i}:$ Emission factor caused by (per unit generated power) by the *i*th generator.

Hence, the total sum of emissions produced by the entire committed unit (power system) is formulated as follows:

4 $$\begin{aligned} \sum _{i=1}^{m}E_{i} = \sum (P_{Gi} \times E_{i}) \end{aligned}$$

Emission pollutants that are produced by fossil-fuelled thermal units, comprising sulphur oxides $SO_x$ and nitrogen oxides $NO_x$, could be analysed independently. However, in order to provide an illustration, the aggregate ton/h emission $E(P_{i})$ among these particles is equal to Eq. (5): as described in^5^ and^8^.

5 $$\begin{aligned} E(P_G)&= \sum _{i=1}^{m} 10^{-2}(\alpha _i + \beta _i P_{Gi} + \gamma _i P_{Gi}^2) + \delta _i \exp (\epsilon _i P_{Gi}) \end{aligned}$$

whereas, $\alpha , \beta , \gamma , \delta$ and $\epsilon$ are various parameters describing different emission coefficients for each generator in the power system.

### Problem constraints

We consider three constraints for the EED problem in our multi-objective optimisation problem i.e. power generation (lower and upper limits), power stability (production meets demand), and security constraints. The mathematical foundations of these constraints are described in subsequent subsections.

#### Power generation constraints

Every single generator’s realistic power output is constrained by its upper and lower limits for reliable operation in the manner described below:

6 $$\begin{aligned} {P_{G}}_{\text {i}}^{\text {min}} \le P_{G_i} \le {P_{G}}_{\text {i}}^{\text {max}}, \quad i = 1, \ldots , M \end{aligned}$$

The generated power at time *t* must be equal to the demand from the total loads side as given below:

7 $$\begin{aligned} \sum _{j=1}^{m} P_{G_{jt}} = P_{D_{load, t}} \end{aligned}$$

#### Power stability constraints

The power stability constraint means that the total amount of generated power $P_{Gi}$ must be equal to the total amount of demand $P_{D}$ plus the actual transmission line losses $P_{loss}$. Hence, mathematically it is given by:

8 $$\begin{aligned} \sum _{i=1}^{m} P_{Gi} = P_{D} + P_{\text {loss}} \end{aligned}$$

In practice, there are numerous ways to determine transmission line losses, including the power flow and the $B_{\text {matrix}}$ technique. Another approach has been used in our implementation, and it entails solving the load flow problem with equality bounds on both reactive and real power at each bus in the way that is described below.

9 $$ P_{Gi} - P_{Di} - V_{i} \sum _{j=1}^{n} V_{j}[G_{ij}\cos (\theta _{i} - \theta _{j}) + B_{ij}\sin (\theta _{i} - \theta _{j})] = 0 $$

10 $$Q_{Gi} - Q_{Di} - V_{i} \sum _{j=1}^{n} V_{j}[G_{ij}\sin (\theta _{i} - \theta _{j}) + B_{ij}\cos (\theta _{i} - \theta _{j})] = 0 $$

where *i* refers to a particular bus index, designating the bus at where power is produced $(P_{Gi})$, consumed $(P_{Di})$, or where the magnitude of the voltage $(V_{i})$ and phase angle (*i*) are obtained. However, *j* indicates an additional bus index that designates a different bus in the network. The equations take into account the contributions from many buses $\text {(from } j = 1 \text { to } j=n)$, as indicated by the summation $(\sum )$ over *j*, where *n* represents the total number of buses in the system. Subsequently, $P_{G_i}$ indicates the power generated by source *i*, $P_{Di}$ is the power consumed by load *i*, $V_{i}$ is the voltage magnitude at bus *i*, $G_{ij}$ is the conductance between buses *i* and *j*. Similarly, $B_{ij}$ determines the susceptance between buses *i* and *j*. Eventually, $\theta _{i}$ and $\theta _{j}$ illustrates the voltage phase angles at buses *i* and *j*, respectively. Thus, the actual power transmission losses can be measured with the following equation.

11 $$\begin{aligned} P_{\text {loss}} = \sum _{k=1}^{N} g_k \left[ V_i^2 + V_j^2 - 2V_iV_j \cos (\theta _i - \theta _j) \right] \end{aligned}$$

In this equation, the power loss can be calculated by combining the inputs of every element (indexed by *k*). Moreover, $g_{k}$ is used to denote the electrical conductance within the *k*th line connecting bus *i* to bus *j*, and *N* represents the total number of transmission lines.

#### Security constraints

Security and integrity constraints can be expressed empirically to define the limits or requirements that must be met for safe and reliable operation. Hence, the transmission line loading *S* is constrained by its upper limit for secure operation as follows:

12 $$\begin{aligned} |S_{li}| \le S_{\text {max}_{li}}, \quad i = 1, 2, \ldots , N \end{aligned}$$

It is important to operate any generator within its minimum and maximum capacity. This should be noted that going above the capacity limit will compromise the system’s security, reliability, and dependability.

### Problem formulation

This problem ought to be mathematically represented as a non-linear constrained MOPs by combining both constraints and objectives simultaneously.

$$\text {Minimize} (P_{G}) \text { in terms of:}$$

13 $$\begin{aligned} g(P_G, F(P_G), E(P_G))&= 0 \end{aligned}$$

14 $$\begin{aligned} h(P)&\le 0 \end{aligned}$$

The equality constraint *g* is dependent on the variables $P_G$, $F(P_G)$, and $E(P_G)$. This condition makes sure that specific requirements are fulfilled. However, the parameter *h* is represented as the inequality constraint that is further dependent on *P*, which ensures that some specific requirements are met.

### The concept of multi-objective optimisation

In the real-world, simultaneous optimisation of multiple objective functions occurs in many problems. These functions usually have incommensurate, predominantly opposing, and contradictory objectives. Considering such competing objective functions, multi-objective optimisation produces a set of optimal solutions instead of just one. Numerous solutions are optimal because no one can be said to be superior to any other with regard to all objective functions. Pareto-optimal solutions are exactly these optimal approaches that have been referred to, as discussed in^59^.

The two competing solutions, $x_{1}$ and $x_{2}$, associated with a MOP, can either dominate the other or else, depending on the situation. If the subsequent two conditions are met, a solution $x_{1}$ to a minimising problem dominates $x_{2}$ regardless of compromising clarity:

15 $$\begin{aligned}{} & {} \forall i \in \{1, 2, \ldots , N_{\text {obj}}\}: f_i(x_1) \le f_i(x_2) \end{aligned}$$

16 $$\begin{aligned}{} & {} \exists j \in \{1, 2, \ldots , N_{\text {obj}}\}: f_j(x_1) < f_j(x_2) \end{aligned}$$

In case, any of the preceding conditions are compromised, the outcome $x_{1}$ fails to dominate over the solution $x_{2}$. The non-dominated solution is $x_{1}$ if it dominates the solution $x_{2}$. The Pareto-optimal set is defined as the set of solutions that are non-dominated across the whole search space. The Pareto optimal front is another name for this set.

## The proposed NS-MJPSO*loc* algorithm

### General overview

In recent years, study on evolutionary approaches illustrates that population-based algorithms are well-suited for solving multi-objective optimisation problems. They can also be effectively used to overcome many of the limitations of traditional single objective strategies, including their sensitivity to the Pareto-optimal front’s shape and their requirement of numerous runs to find various Pareto-optimal solutions.

Traditionally, the main goal of a MOP algorithm is to maintain population diversity in the set of Pareto optimal solutions, besides steering the search towards the Pareto-optimal front.

PSO has been inspired as a promising social heuristic method with an adaptable and diverse strategy to improve and modify the capabilities of both local and global exploration in recent years. In contrast, considering that there is not a definite global best in multi-objective PSO, only a set of non-dominated solutions, transforming classical single objective-based to a multi-objective-based PSO necessitates redefining global and local best candidates. Additionally, there could not be a single local best solution with every swarm particle. In a multi-objective space, selecting the global and local best for steering the population turns into a challenging problem.

The challenges of evolving a multi-objective variant of the standard PSO are resolved by the proposed approach, which involves a process for choosing between the global and local best candidates. It is important to note that the suggested MOPSO technique has been applied with remarkable success to a number of challenging standard test problems in the field of multi-objective optimisation^60^.

### Core concepts and notions

The brief descriptions, definitions, and terminologies of the proposed NS-MJPSO*loc* algorithm are given as follows:

- Particle, current position: $X_{i}(t)$ is a candidate solution, where *d* is the total number of optimized parameters, and is represented by an *d*-dimensional vector. $X_{i,d}(t) = [x_{1,d}(t),..., x_{n,d}(t)]$, where $X_{i,d}(t)$ represents the position of the particle *i*th with respect to the dimension *d*, or the value of the dimension parameter *d* in the candidate solution *i*th, describes the particle *i*th at time *t*.
- Swarm size, population: *S*(*t*), represents a distinct set of *n* particles in time *t*, whereas, $S(t) = [x_{1}(t),..., x_{n}(t)]$
- Velocity vector, $V_{i}(t)$: The parameter identified as velocity adjusts how each particle $V_{i,d}(t)$ moves in the *d*-dimensional search space. In order to identify optimal or near-optimum solutions, it coordinates the ways to exploit and explore the swarm in the search space. $V_{i,d}(t) = [v_{1,d}(t),..., v_{n,d}(t)]$, where the parameter *t* represents time.
- *N* states Markov Jumping: It is a mathematical illustration of a series of occurrences or states where the next state is solely dependent upon the current state. Furthermore, it relates to a particular type of Markov chain with a set of states represented by *N*.
- Evolutionary factor, $E_{f}$: In order to automatically adjust the inertia weight and acceleration coefficients, an evolutionary factor was developed that determines four specific evolutionary stages such as convergence, exploitation, exploration, and jumping out in each generation^61–65^. The evolutionary factor is able to consider data about population distribution. In this paper, we define four states using the evolutionary factor. The following expression represents the mean distance of each particle in the whole swarm:
  17 $$\begin{aligned}{} & {} P_{d}(i)= \frac{1}{N-1}\sum _{j=1,j\ne i}^{N}\sqrt{\sum _{k=1}^{D}(x_{i}(k)-\bar{x}_{j}(k))^2} \end{aligned}$$
  18 $$\begin{aligned}{} & {} E_{f}= \frac{P_{dg}-P_{d(min)}}{P_{d(max)}-P_{d(min)}}\in [0,1] \end{aligned}$$
  wherein Eq. (17), *S* represents population size and *D* represents the dimension of the search space and Eq. (18), $P_{dg}$ denotes the global best particle among the $P_{d}(i)$, $P_{d(min)}$ and $P_{d(max)}$ are particles with minimum and maximum distances, respectively.
- Inertia weight, $\omega (t)$: The control parameter known as inertia weight, or $\omega (t)$, serves to determine how significantly the preceding velocities influence the current velocity. As a result, it influences the balance between the global and local exploring capacities. A large inertia weight is recommended for the early stages of the search process to improve global exploration, however, a smaller inertia weight is proposed for the later stages to improve local exploration. It is assumed that $E_{f}$ is significantly large in the jump state and small in the convergence state. The evolutionary factor $E_{f}$ and the inertia weight $\omega$ share several characteristics. As a result, the mapping $\omega (E_{f})$ is defined as follows:
  19 $$\begin{aligned} \omega (E_{f})= 0.5E_{f} + 0.4 \in [0.4,0.9], \forall E_{f}[0,1] \end{aligned}$$
- Neighbourhood $K_{i}$, *Lbest*: A subgroup of neighbouring particles, labelled $K_{i}$, this group is known as the neighbourhood of particle *i*. Based on the neighbourhood’s topology (such as a ring or a star), its size can be determined. Within its defined neighbourhood search space, $K_{i}$ indicates the indexes of neighbouring particles for each particle *i*. Considering the *Lbest* strategy, the velocity update model for particle *i* at time $t+1$ can be expressed as follows:
  20 $$\begin{aligned}{} & {} v_{i}(t+1) = \omega v_{i}(t) + c_{1}(\delta (t))r_{1}(t)(Pbest_{i}(t) - x_{i}(t)) + c_{2}(\delta (t))r_{2}(t)(Lbest_{i}(t) - x_{i}(t)) \end{aligned}$$
  21 $$\begin{aligned}{} & {} x_{i}(t+1) = x_{i}(t)+ v_{i}(t+1) \end{aligned}$$
  Whereas, the $Lbest_i(t)$ indicates the particle’s best position with the best fitness value observed in its immediate neighbourhood.

### Computational stream

In the computational steam of the proposed NS-MJPSO*loc* algorithm, we have *n* number of particles with *D* dimensional parameters and neighbourhood $K_{i}$. It can be described in the following Algorithm 1, Algorithm 2, and Algorithm 3.

**Algorithm 1:**
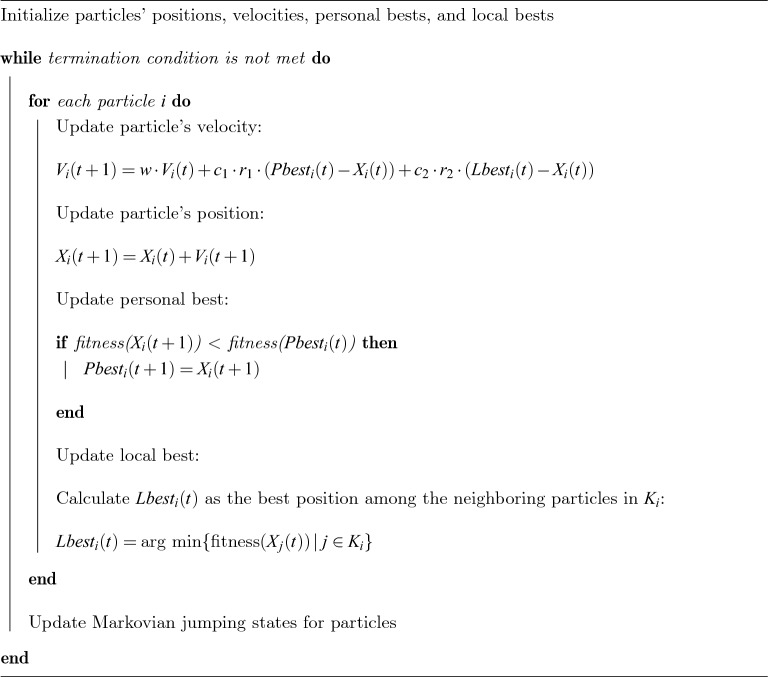
Neighbourhood-aware strategy.

**Algorithm 2:**
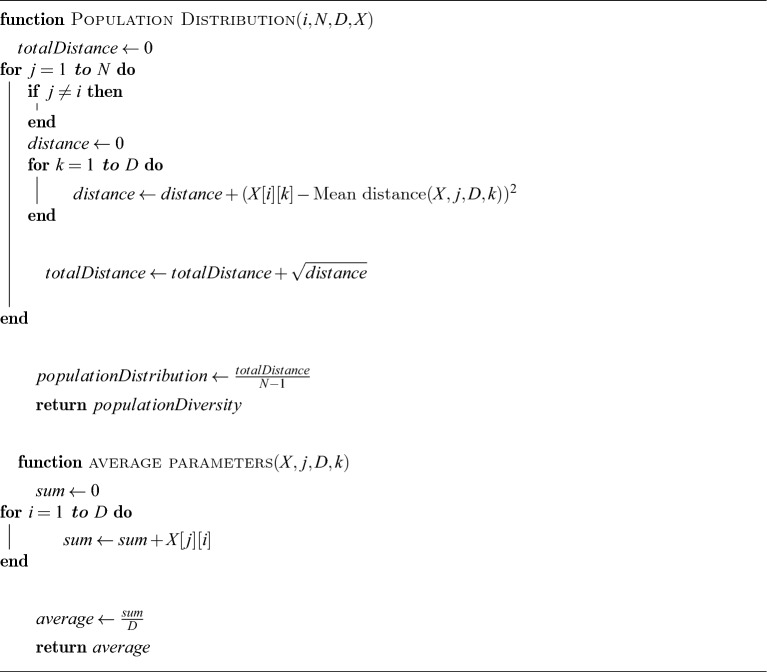
Population Diversity Measure.

**Algorithm 3:**
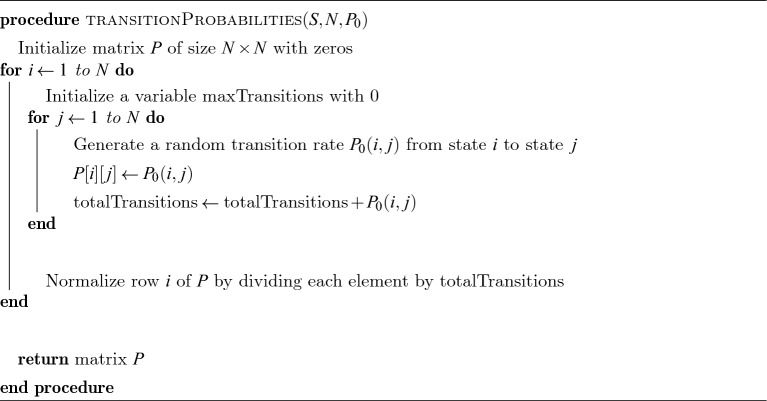
Markov Transition Probability Matrix.

### The mechanism of selecting acceleration coefficients

**Figure 1:**
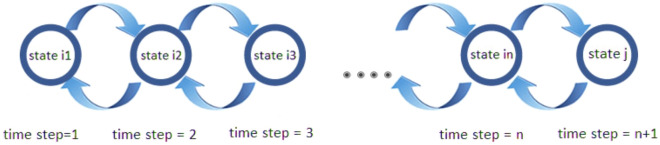
Illustration of the the jumping-out state.

In this work, the acceleration coefficients $c_{1}(\delta (0))$ and $c_{2}(\delta (0))$ with initial values of 2 are discussed in this study. Then, on the basis of the evolutionary state, these coefficients are automatically updated in the following phases.

**Figure 2:**
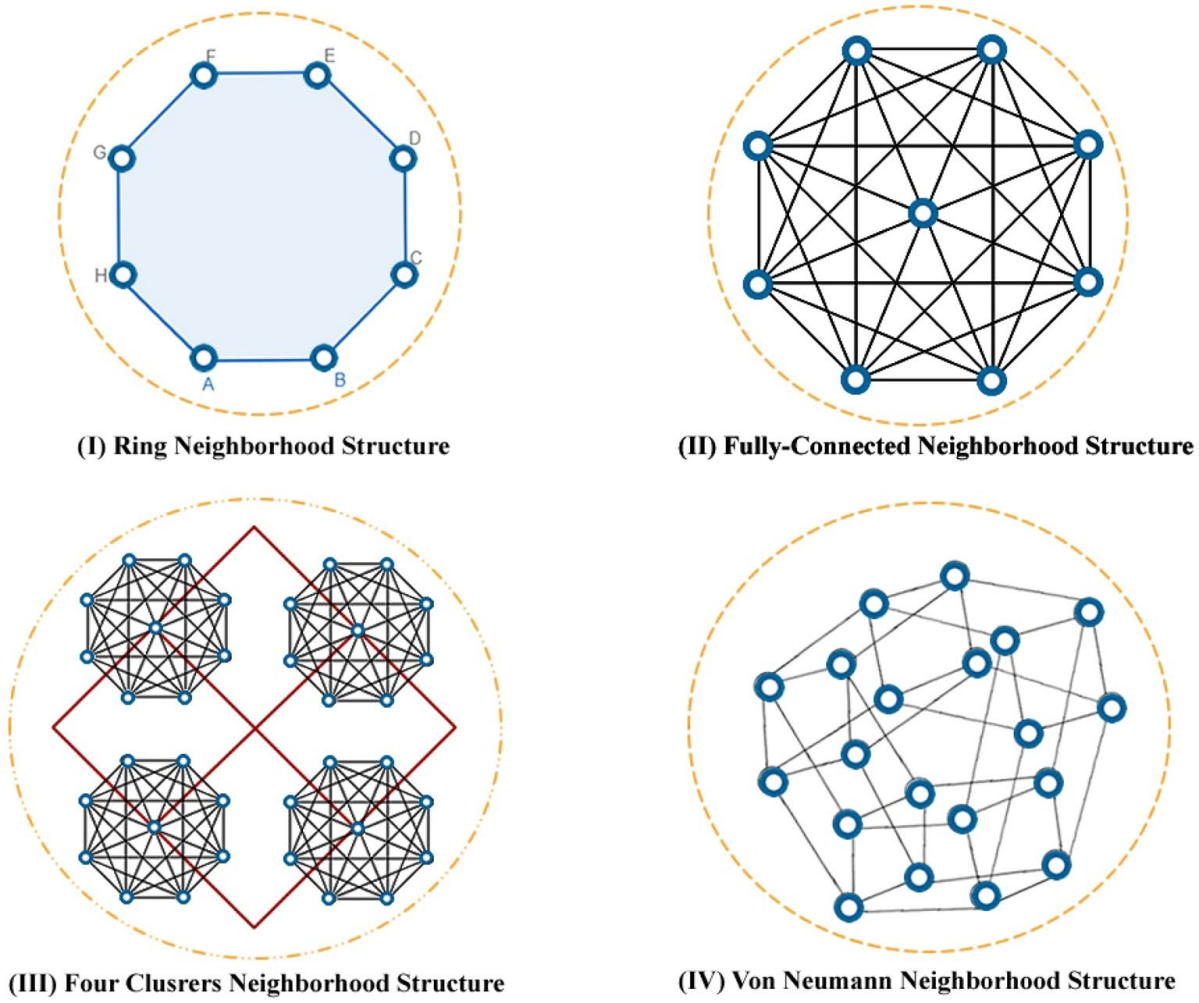
The neighbourhood’s topology using four different structures.

- **Jumping-out-state** The primary goal of the jumping-out state is to allow the particles to escape local optima and get closer to a more advantageous global optima. The acceleration coefficients $c_{1}$ and $c_{2}$ are adjusted appropriately to enable this operation. Figure 1 shows the Markov switching based on the transition probability. Usually, a higher value of $c_{2}$ and a lesser value of $c_{1}$ are employed in this phase. These changes are made to encourage particles to move more quickly in the direction of the best particle overall. A greater $c_{2}$ accelerates convergence by amplifying the effects of the particle’s individual best position and the global best position. However, a smaller $c_{1}$ minimises the influence of the particle’s previous velocity, enabling it to more successfully explore new regions. The values for $c_{1}$ and $c_{2}$ in the aforementioned case are set to $c_{1}(\delta (4))$ = 1.8 and $c_{2}(\delta (4))$ = 2.2, respectively. These parameters are selected to allow escape from local optima and to encourage quick progress towards the particle that performs best globally.
- **Exploration state** In the exploration state, the focus is on analysing new optima while avoiding getting stuck in existing ones. In this phase, $c_{1}$ is used with a greater value, whereas $c_{2}$ is used with a relatively smaller value. By favouring the past velocity of the particle $c_{1}$ over the impact of the global best position $c_{2}$, these changes aim to promote individual exploration. The values for $c_{1}$ and $c_{2}$ in the precise instance are set at $c_{1}(\delta (3))$ = 2.2 and $c_{2}(\delta (3))$ = 1.8, respectively. By letting the particle rely more on its own velocity to explore new areas of the search space, these values are set to encourage individual exploration. To establish a balance between exploration and exploitation, acceleration coefficients are frequently dynamically adjusted throughout the optimisation process.
- **Exploitation state** The main purpose of the exploitation phase is to employ the local knowledge that the particles have while looking for the best solutions in the potential region. Typically, this state comes after the exploration state and before the convergence state. In this state, $c_{1}$ serves a substantially bigger value, whereas $c_{2}$ is used with a relatively smaller value. With these adjustments, the particle’s own best position (local information) will be given more weight, but the impact of the global best position will still be taken into account. The values for $c_{1}$ and $c_{2}$ in the precise instance are set at $c_{1}(\delta (2))$ = 2.1 and $c_{2}(\delta (2))$ = 1.9, respectively. These values were selected to strike a compromise between using each particle’s local information and investigating the prospective region as a whole.
- **Convergence state** Finally, in the convergence state, the swarm has a tendency to form dense clusters and become close to the overall best solution. The proposed neighbourhood’s topology is shown in Fig. 2. However, there is a chance of early convergence, in which the particles may become trapped in less-than-ideal solutions and stop further exploring the search space. The acceleration coefficients $c_{1}$ and $c_{2}$ are modified to address this problem and maintain search diversity. The values for $c_{1}$ and $c_{2}$ in the exact scenario are set at $c_{1}(\delta (1))$ = 2 and $c_{2}(\delta (1))$ = 2, respectively, in the convergence stage. These parameters have been selected to promote exploration and preserve search diversity within the swarm, while also pointing the particles in the direction of the present global area. The particles strike a compromise between exploration and exploitation by setting $c_{1}$ and $c_{2}$ to the same value. This method enables the swarm to carry on searching and maybe find better solutions, preventing premature convergence.

## Performance evaluation

### Experimental setup

In this paper, the goal was to effectively solve nonlinear constrained optimisation problems using the NS-MJPSO*loc* algorithm. In order to do this, an additional method of neighbourhood awareness (as shown in Fig. 2) was added to the proposed algorithm to evaluate the viability of potential solutions at each stage of the search. This process is used in the study to guarantee that the non-dominated solutions are both optimal and compliant with the set of constraints. The proposed NS-MJPSO*loc* algorithm can successfully handle a challenging optimisation problem with several objectives given various constraints. Table 4 describes various experimental parameter settings for all compared algorithms.

**Table 4:** Parameter settings for all the compared algorithms.

Algorithm	Parameter
GA	$N = 50, cp=0.01, mp=0.05$
EP	$N = 50, l_{rate}$
DE	$N = 50$
PSO	$N = 50, c_{1}, c_{2} = 1.9, w = 0.9$
NS-MJPSO	$N = 50, N-States = 4,\phi =0.9$
NS-MJPSO*loc*	$N = 50, Neighbourhoods = 6, w = 0.9 \phi = 0.9, c_{1} = \{1.8, 2.2, 2.1, 2 \}$
	$$\hbox {c}_{2} = \{2.2, 1.8, 1.9, 2 \}$$

A desktop PC Core*i*5 with 3.30*GHz*, 8GB RAM, Windows 10 Enterprise was used to carry out the evaluation of the proposed NS-MJPSO*loc* approach. Furthermore, the MATLAB R2018b application is used for algorithm development, data analysis, visualization and production of results. A collection of parameters from Table 4 with generators data in Table 5 was used during the optimisation runs.

**Table 5:** Generator data^66^.

Generator no.	Min. (MW)	Max. (MW)	a ($/MW^2^h)	b ($/MW h)	c ($/h)
1	50	200	0.003750	2.000	0
2	20	80	0.017500	1.700	0
3	15	50	0.062500	1.000	0
4	10	35	0.008340	3.250	0
5	10	30	0.025000	3.000	0
6	12	40	0.025000	3.000	0

**Figure 3:**
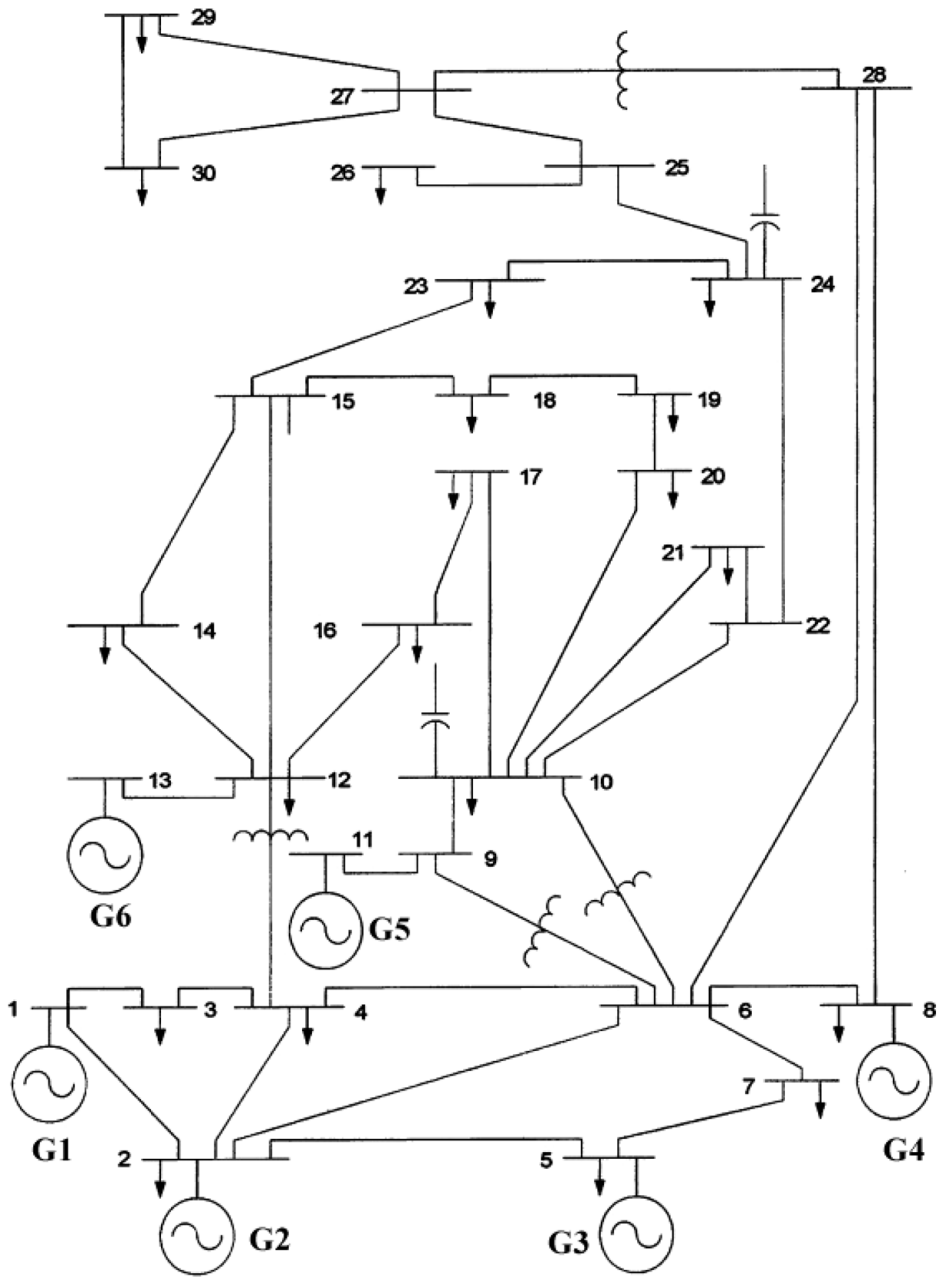
IEEE 30-bus test system^63^.

The maximum number of generations was established at 1000, the number of particles was decided to be 100 and the tests were repeated 30 times. The results shown in various tables are averaged over these multiple runs. The datasets used in the experiments were taken from previous published sources^63^ and online open source code repositries (https://github.com/P-N-Suganthan). Furthermore, the power system, bus unit, and other datasets generated and/or analysed during the current study are publicly available in the Github repository, and can be accessed at (https://github.com/evgenytsydenov/ieee118_power_flow_data). Moreover, various IEEE bus systems for power systems used within the experimental work are publicly available online.

A threshold of 25 solutions was placed in place to keep the Pareto-optimal set at a suitable size. The neighbourhood-best set has a maximum size of 10 solutions as well. In order to manage and regulate the size of these solution sets, a neighbourhood strategy is used if the number of non-dominated Pareto-optimal solutions in either the global best set or the neighbourhood-best set exceeds their respective boundaries.

### Evaluation metrics

The proposed algorithm is compared with other closest rivals using several performance evaluation metrics such as fuel costs (measured in US dollars per hour), electricity/power generation through each generator (MW), carbon emission (measured in tons per hour), and computational time (measured in seconds).

### Results and discussion

The proposed NS-MJPSO*loc* technique was used for the first time on the typical IEEE 30-bus 6-generator test system to assess its efficacy. This system is frequently referenced in the literature, and numerous strategies have already been tried on it with promising results.

Figure 3 shows the single-line diagram of the IEEE test system, and references^5,8^ provide in-depth information about the system. Table 5 lists the values of the fuel costs and the emission coefficients related to the generators.

Three cases with different levels of complexity were taken into account in order to demonstrate the efficacy of the proposed algorithm. These examples were chosen to show how well the algorithm performed under various conditions and tasks.

**Table 6:** Method Comparison with closest rivals.

Method	Penalty factor ($/lb)	P1 (MW)	P2 (MW)	P3 (MW)	P4 (MW)	P5 (MW)	P6 (MW)	Total fuel cost ($/h)
GA	3.480	58.413	76.270	47.826	33.448	28.759	39.980	2107.195
EP	3.480	121.565	156.582	36.303	28.910	22.880	21.900	2122.521
DE	3.650	120.324	152.875	34.34	27.12	21.19	20.789	2112.530
PSO	3.480	104.730	46.600	27.930	35.000	30.000	40.000	2109.470
NS-MJPSO	3.480	101.565	126.582	34.303	27.910	25.880	25.432	2087.786
NS-MJPSO*loc*	3.480	94.290	55.380	30.190	32.780	29.360	30.660	2079.390

- **With** $P_{Loss}$: In this scenario, transmission losses $P_{loss}$ are considered along with power balancing and generation capacity constraints.
- **Without** $P_{Loss}$: Here transmission losses $P_{loss}$ are ignored, while power balancing and generation capacity constraints are taken into consideration.
- **All constraints:** In this case, all relevant constraints were considered as described in the problem constraints, i.e., Section Problem constraints.

The aim of this study is to provide a smart solution to the ELD problem with line flow and emission limits. Considering the IEEE 30-bus system, the EED problem in power systems is investigated and evaluated with several plausible assumptions. Furthermore, to achieve the EED schedule with the least amount of generation and cost of the generating units, this paper uses a variety of PSO variants, such as GA, EP, PSO, and DE. The performance of a newly developed PSO variant i.e., NS-MJPSO*loc* is also investigated and compared with other methods. The IEEE 30-bus system’s generating characteristics are listed in Table 5.

The study rendered employing a variety of intelligent algorithms mentioned earlier, and the results of the transitional cost analysis for the IEEE 30-bus system are shown in Table 6. The results shown that our proposed NS-MJPSO*loc* technique can approximately 2.3%, 3.0%, 2.5%, 2.4%, and 0.4% fuel costs per hour as compared to GA, EP, DE, PSO, and NS-MJPSO techniques, respectively. Tables 7, 8, 10 also show the IEEE 30-bus system’s convergence criteria, given that line flow constraints are taken into consideration. Table 10 compares the minimal total production costs attained utilizing the above algorithms for a demand of 283.4 MW.

**Table 7:** Algorithm Performance on IEEE 30 Bus and 15-unit Systems.

Algorithm	GA	EP	DE	PSO	NS-MJPSO	NS-MJPSO*loc*
Population size (NP)	10/10	10/10	10/10	10/10	10/10	10/10
Chromosome length (bits)	20/20	–	–	–	–	–
Max. iterations	500/4000	500/4000	500/4000	500/4000	100	100
Crossover probability (cP)	0.895/0.895	–	–	0.895/0.895	–	–
Mutation probability (mP)	0.0053/0.008349	–	–	–	–	–
Scaling factor, transition prob ($\beta , \phi$)	–	0.008/0.0016	–	1.9/1.9	0.9/0.9	0.9/0.9
Time taken (s) for the IEEE 30 Bus system	27.970	16.344	15.703	15.469	14.54	14.27
Time taken (s) for the 15-unit system	35.605	18.063	14.609	20.235	16.32	12.02

**Table 8:** Generator characteristics of 15-unit systems.

Unit	$$P_{\text {min}_i}$$(MW)	$$P_{\text {max}_i}$$(MW)	$$a_i$$($/MW^2^h)	$$b_i$$($/MWh)	$$c_i$$($/h)
1	150	455	0.000299	10.1	671
2	150	455	0.000183	10.2	574
3	20	130	0.001126	8.8	374
4	20	130	0.001126	8.8	374
5	150	470	0.000205	10.4	461
6	135	460	0.000301	10.1	630
7	135	465	0.000364	9.8	548
8	60	300	0.000338	11.2	227
9	25	162	0.000807	11.2	173
10	25	160	0.001203	10.7	175
11	20	80	0.003586	10.2	186
12	20	80	0.005513	9.9	230
13	25	85	0.000371	13.1	225
14	15	55	0.001929	12.1	309
15	15	55	0.004447	12.4	323

**Figure 4:**
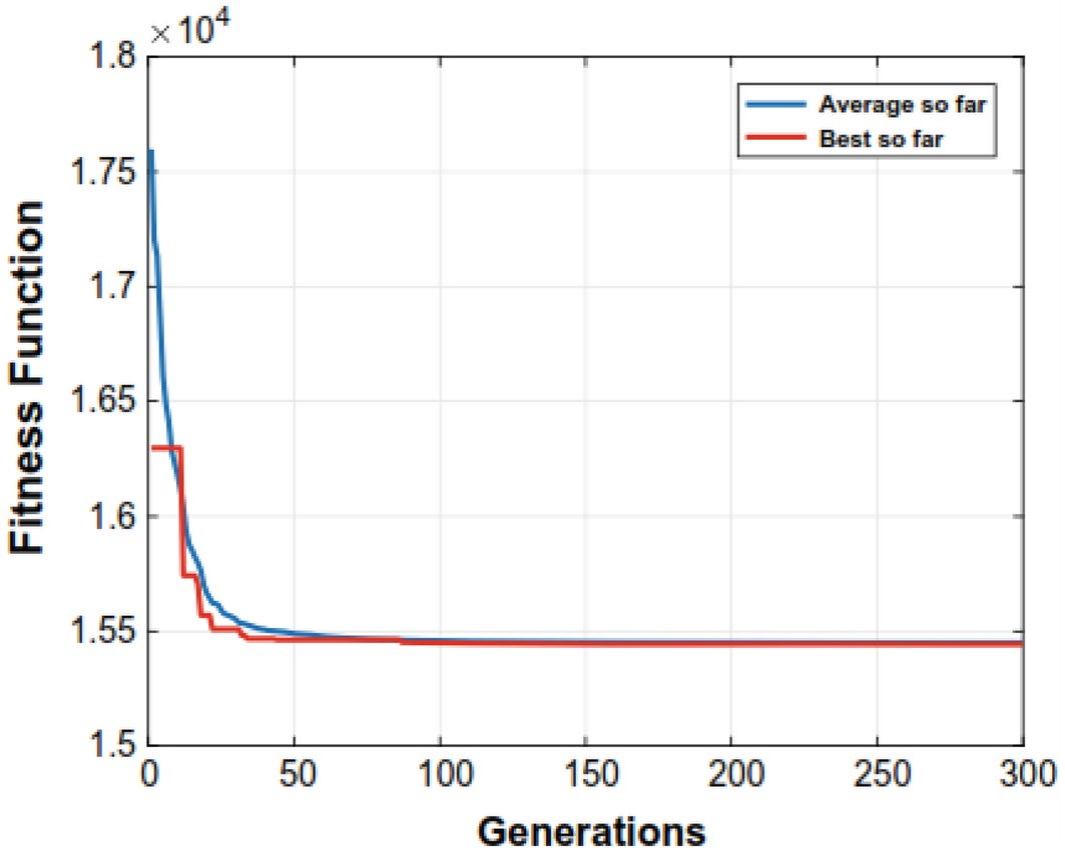
Convergence of the proposed algorithm over a 6-unit system.

**Figure 5:**
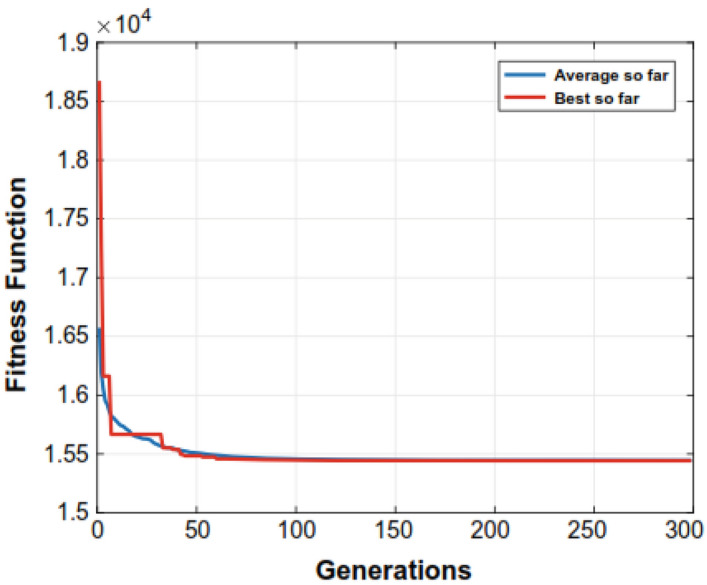
Convergence of the DE evolutionary process throughout a 6-unit system.

**Figure 6:**
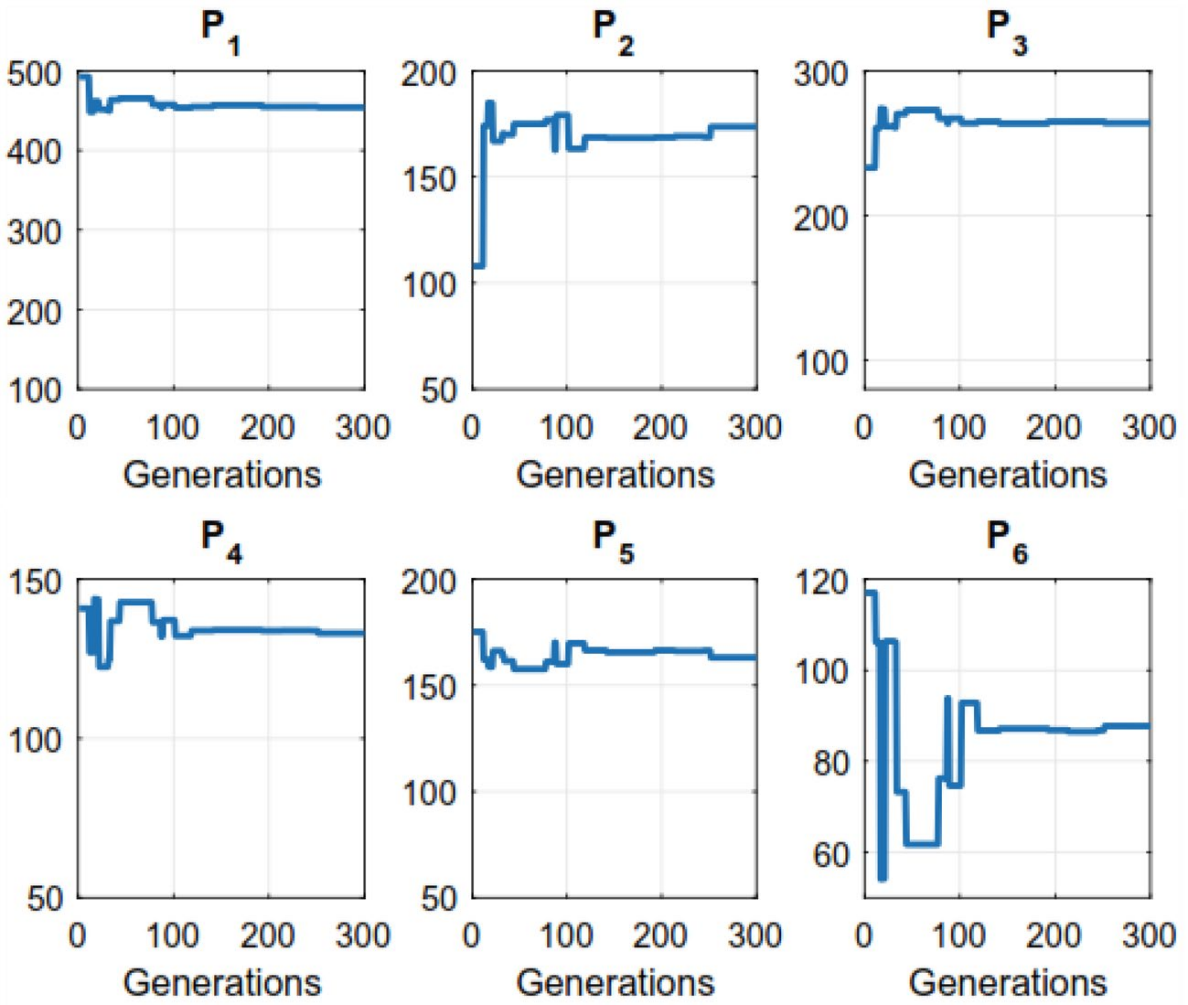
Results of NS-MJPSO*loc* optimal dispatch load analysis for the 6-unit power system.

**Figure 7:**
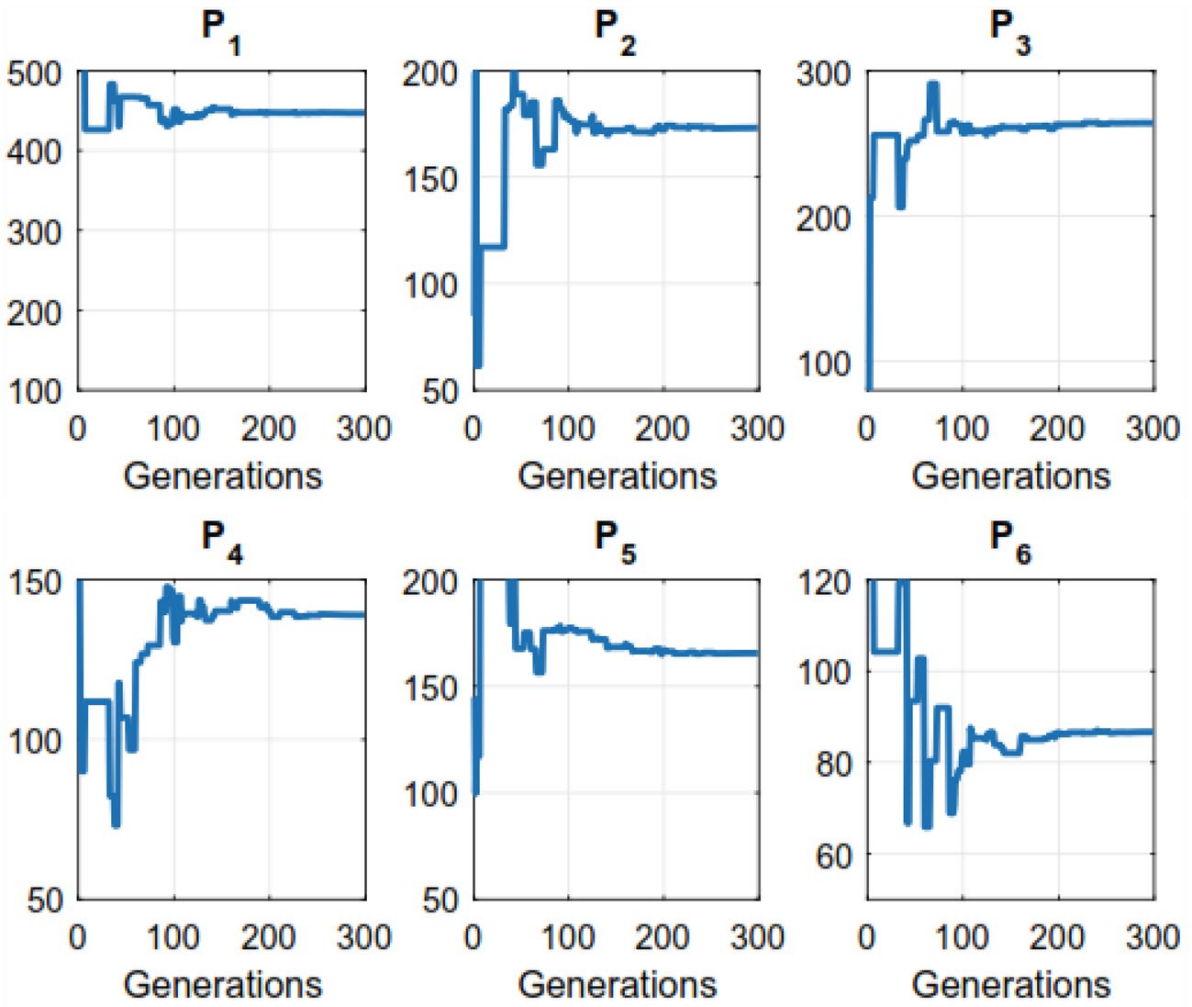
Using DE, the determined optimal dispatch load for a 6-unit power system.

The data summarized in Table 6 shows that, when compared to the overall minimal production costs obtained by using (EP) technique, the costs obtained in the research are noticeably higher. The (EP) algorithm requires more generations to reach convergence than the other techniques (GA, PSO, and DE). This shows that finding the best solution for (EP) might require additional iterations.

The (GA) needs additional solution time compared to the other methods evaluated in the research. The parameters that the aforementioned methods such as (GA, EP, PSO, and DE) apply at various times are detailed in Table 7. We observed that the proposed algorithm could save significant computational times as compared to other closest rivals. For example, for a 15-unit bus system, NS-MJPSO*loc* runs approximately 49%, 12.7%, 9.1%, 7.8%, and 1.9% more efficiently than GA, EP, DE, PSO, and NS-MJPSO algorithms, respectively. Similarly, these values were noted as 66.2%, 33.5%, 17.7%, 40.6%, and 26.3% for a 30-unit bus system. The effectiveness and efficiency of each method in solving the ELD problem are greatly influenced by these parameters. Figures 4 and 5 show the convergence rates of the proposed NS-MJPSO*loc*’s and DE’s evolutionary processes throughout a 6-unit bus system, respectively. Furthermore, Figs. 6 and 7 show the results of the NS-MJPSO*loc*’s and DE’s optimal dispatch load analysis for the 6-unit power system, respectively.

Moreover, the study shows that the (EP) technique works better than other algorithms in terms of reaching a lower overall production cost for the specified demand. To achieve convergence, though, more generations must be produced. The (GA), in contrast, takes longer to solve problems than the other methods. The parameter settings of each method are presumably revealed in Table 4, which explains why each algorithm performed differently in the article.

**Table 9:** Comparison of ELD with line flow constraints using different intelligent techniques for the 15-unit system.

Intelligent Techniques	GA	EP	DE	PSO	NS-MJPSO	NS-MJPSO*loc*
$$P_1$$ (MW)	455	283.6025	219.4531	168.9527	142.3432	144.8345
$$P_2$$ (MW)	303.7664	151.6028	150.0000	150.0000	135.5649	132.8716
$$P_3$$ (MW)	75.4567	130.0000	130.0000	130.0000	197.2647	171.7489
$$P_4$$ (MW)	75.4567	130.0000	130.0000	130.0000	191.3441	158.3831
$$P_5$$ (MW)	311.3287	236.9869	183.4387	150.0000	181.8172	147.5173
$$P_6$$ (MW)	298.8495	460.0000	460.0000	460.0000	450.2432	448.3729
$$P_7$$ (MW)	301.3702	305.1626	465.0000	465.0000	453.7198	445.2437
$$P_8$$ (MW)	180.9965	257.3319	216.2897	300.0000	298.1379	288.1428
$$P_9$$ (MW)	94.0688	162.0000	162.0000	162.0000	160.1455	157.1319
$$P_{10}$$ (MW)	93.0605	160.0000	160.0000	160.0000	158.1278	152.2912
$$P_{11}$$ (MW)	50.2491	79.9141	80.0000	80.0000	77.1489	74.8733
$$P_{12}$$ (MW)	50.2491	80.0000	80.0000	80.0000	77.1274	73.2539
$$P_{13}$$ (MW)	55.2491	85.0000	85.0000	85.0000	80.1182	82.2479
$$P_{14}$$ (MW)	35.166097	55.0000	55.0000	55.0000	54.2567	52.4653
$$P_{15}$$ (MW)	35.166097	55.0000	55.0000	55.0000	53.1444	50.2381
Fuel cost ($/h)	64046.5172	64246.000	66993.000	68231.000	63901.000	63855.000

**Table 10:** The optimal solutions for cost and emission are optimized separately (in terms of fuel costs and carbon emissions).

	Case 1		Case 2		Case 3	
	Cost	Emission	Cost	Emission	Cost	Emission
$$PG_1$$	0.1189	0.4016	0.1176	0.4128	0.1553	0.4511
$$PG_2$$	0.2976	0.4556	0.3187	0.4617	0.3401	0.5167
$$PG_3$$	0.5265	0.5380	0.5921	0.5499	0.7811	0.6534
$$PG_4$$	1.1198	0.3852	0.9825	0.3843	1.0123	0.4387
$$PG_5$$	0.5222	0.5363	0.5112	0.5498	0.1031	0.1923
$$PG_6$$	0.3527	0.5143	0.3512	0.5123	0.4738	0.6167
Cost (US$/h)	600.1100	638.2400	607.7800	645.2300	618.4800	656.7300
Emission (ton/h)	0.2221	0.1942	0.2198	0.1942	0.2302	0.2013

**Table 11:** Solutions for cost and emission optimized using multi-objective.

	Best cost			Best emission		
	Case 1	Case 2	Case 3	Case 1	Case 2	Case 3
$$PG_1$$	0.1183	0.1207	0.1524	0.4015	0.4101	0.4589
$$PG_2$$	0.3019	0.3131	0.3427	0.4590	0.4594	0.5121
$$PG_3$$	0.5224	0.5907	0.7857	0.5332	0.5511	0.6524
$$PG_4$$	1.0116	0.9769	1.0180	0.3891	0.3919	0.4331
$$PG_5$$	0.5254	0.5155	0.0995	0.5456	0.5413	0.1981
$$PG_6$$	0.3544	0.3504	0.4669	0.5057	0.5111	0.6129
Cost (US$/h)	600.12	607.79	618.54	637.42	644.74	656.87
Emission (ton/h)	0.2216	0.2193	0.2308	0.1942	0.1942	0.2014

The generator attributes for the 15-unit system are presented in Table 8. Furthermore, Table 9 provides an overview of the comparison of ELD with line flow constraints using different intelligent techniques for a 15-unit system. We observed that our approach can reduce $\sim$ 6.4% of fuel costs in comparison to the classical PSO technique. Furthermore, approximately 0.3%, 0.61%, 4.7%, and 0.07% fuel costs can be saved by the proposed NS-MJPSO*loc* techniques in comparison to GA, EP, DE, and NS-MJPSO techniques, respectively. Table 10 represents the actual power generation output of the 15-unit system and the transition cost of the 15-unit system, determined by a variety of intelligent techniques. Table 7 demonstrates the convergence conditions for the 15-unit system concerning emission and line flow constraints using GA, EP, PSO, and DE. A breakdown of the minimum total production costs for demand of 2630 MW using smart techniques like GA, EP, PSO, and DE is shown in Table 10. A description of the cost estimation is given in the Smooth cost minimization section. As reported in Table 10, our method can reduce approximately 9.4% of the emissions measured in tons per hour as compared to the PSO approach.

The results in Table 7 to Table 8 show that for both the IEEE 30-bus system and the 15-unit systems, the PSO algorithm exhibits rapid convergence and requires less time. Although GA requires more time for convergence, it outperforms other intelligent techniques for the 15-unit system when the total minimum generation cost is taken into account. Table 11 shows the solutions for cost and emission optimized using the proposed multi-objective algorithm.

## Conclusions and future work

In this paper, the environmental/economic power dispatch (EED) optimisation problem was addressed using a newly developed, neighbourhood-aware, n-states Markovian jumping PSO (NS-MJPSO*loc*) algorithm. The (NS-MJPSO*loc*) algorithm is a variant of the conventional PSO approach that was created specifically to handle MOPs. The fuel cost and environmental impact were two competing objectives, taken into account when formulating the EED problem. The results derived show the significant potential and effectiveness of the proposed method (NS-MJPSO*loc*) in solving the multi-objective EED problem. The method also demonstrated a significant capacity to generate a variety of evenly distributed solutions within the non-dominated set. While comparing the simulation results, it was obvious that the (NS-MJPSO*loc*) method outperformed other variants of the PSO algorithm in the diversity and quality of the Pareto-optimal solutions achieved. These findings indicate that the proposed method holds promise in enhancing the optimisation process and facilitating better decision-making in power dispatch optimisation, considering both economic and environmental aspects. The evaluation of the proposed approach demonstrated that it can reduce $\sim$6.4% of fuel costs and $\sim$9.1% of computational time, and $\sim$9.4% of the emissions measured in tons per hour, in comparison to the classical PSO technique.

The following are limitations and potential future directions for this research.

The primary limitation of the proposed version is the complexity is increased exponentially as compared to the simplified framework of the PSO algorithm. The (NS-MJPSO*loc*) algorithm could potentially be further improved and optimized to increase its effectiveness and efficiency in resolving multi-objective EED problems. Robust mechanisms for the adjustment of various parameters, other neighbourhood topologies, dynamic adaptive mechanisms, constraints handling, multi-objective optimisation metrics, and merging with other algorithms. By following these research directions, the (NS-MJPSO*loc*) algorithm can be further enhanced, and its adaptability to different scenarios can be improved.

Implementing the proposed approach in a real-world power dispatch environment would provide useful information about its usefulness and efficacy. Furthermore, the incorporation of renewable energy sources into power networks expands the EED problem formulation to consider these sources and their irregular role. This would overcome the hassle created by the integration of renewable energy sources and make the optimisation procedure more analogous to contemporary power systems. Ultimately, these efforts can contribute to more effective and reliable solutions for multi-objective EED optimisation problems, advancing sustainable energy management practices in power systems. In the future, we will extend this work to an effective energy management system within the domain of smart grids.

## Data Availability

The datasets generated and/or analysed during the current study are publicly available in the Github repository, and can be accessed at [https://github.com/evgenytsydenov/ieee118_power_flow_data]. Moreover, various IEEE bus systems for power systems used within the experimental work are publicly available online.

## References

[1] El-Keib A, Ma H, Hart J (1994). Economic dispatch in view of the clean air act of 1990. IEEE Trans. Power Syst..

[2] Talaq J, El-Hawary F, El-Hawary M (1994). A summary of environmental/economic dispatch algorithms. IEEE Trans. Power Syst..

[3] Heslin JS, Hobbs BF (1989). A multiobjective production costing model for analyzing emissions dispatching and fuel switching (of power stations). IEEE Trans. Power Syst..

[4] Granelli G, Montagna M, Pasini G, Marannino P (1992). Emission constrained dynamic dispatch. Electr. Power Syst. Res..

[5] Farag A, Al-Baiyat S, Cheng T (1995). Economic load dispatch multiobjective optimization procedures using linear programming techniques. IEEE Trans. Power Syst..

[6] Dhillon J, Parti S, Kothari D (1993). Stochastic economic emission load dispatch. Electr. Power Syst. Res..

[7] Chang C, Wong K, Fan B (1995). Security-constrained multiobjective generation dispatch using bicriterion global optimisation. IEE Proc.-Gen. Transm. Distrib..

[8] Yokoyama R, Bae SH, Morita T, Sasaki H (1988). Multiobjective optimal generation dispatch based on probability security criteria. IEEE Trans. Power Syst..

[9] Han, G., Xia, Y. & Min, W. Micro-grid environmental economic dispatch using improved linearly decreasing weight particle swarm optimization. In *Mechatronics and Automatic Control Systems: Proceedings of the 2013 International Conference on Mechatronics and Automatic Control Systems (ICMS2013)* 491–500 (Springer, 2014).

[10] Tlijani K, Guesmi T, Abdallah HH (2016). Extended dynamic economic environmental dispatch using multi-objective particle swarm optimization. Int. J. Electr. Eng. Inf..

[11] Taheri B, Aghajani G, Sedaghat M (2017). Economic dispatch in a power system considering environmental pollution using a multi-objective particle swarm optimization algorithm based on the pareto criterion and fuzzy logic. Int. J. Energy Environ. Eng..

[12] Manojkumar, T. & Singh, N. A. Solution of environmental/economic (eed) power dispatch problem using particle swarm optimization technique. In *2018 International Conference on Control, Power, Communication and Computing Technologies (ICCPCCT)* 347–351 (IEEE, 2018).

[13] Lu K-D, Wu Z-G (2022). Multi-objective false data injection attacks of cyber-physical power systems. IEEE Trans. Circuits Syst. II Express Briefs.

[14] Kumar TM, Singh NA (2019). Environmental economic dispatch with the use of particle swarm optimization technique based on space reduction strategy. Eng. Technol. Appl. Sci. Res..

[15] Xin-gang Z, Ze-qi Z, Yi-min X, Jin M (2020). Economic-environmental dispatch of microgrid based on improved quantum particle swarm optimization. Energy.

[16] Mehrpour, M., Seyedi, I. & Askari, M. Dynamic economic load-emission dispatch in power systems with renewable sources using an improved multi-objective particle swarm optimization algorithm. In *2020 2nd International Conference on Electrical, Control and Instrumentation Engineering (ICECIE)*, 1–9 (IEEE, 2020).

[17] Kumar, D., Jain, N. & Nangia, U. Combined economic emission dispatch using perfectly convergent particle swarm optimization. In *2022 IEEE Delhi Section Conference (DELCON)*, 1–10 (IEEE, 2022).

[18] Srinivasan D, Chang C, Liew A (1994). Multiobjective generation scheduling using fuzzy optimal search technique. IEE Proc.-Gen. Transm. Distrib..

[19] Huang C-M, Yang H-T, Huang C-L (1997). Bi-objective power dispatch using fuzzy satisfaction-maximizing decision approach. IEEE Trans. Power Syst..

[20] Das DB, Patvardhan C (1998). New multi-objective stochastic search technique for economic load dispatch. IEE Proc.-Gen. Transm. Distrib..

[21] Abido M (2003). A novel multiobjective evolutionary algorithm for environmental/economic power dispatch. Electr. Power Syst. Res..

[22] Abido MA (2003). Environmental/economic power dispatch using multiobjective evolutionary algorithms. IEEE Trans. Power Syst..

[23] Chen M-R, Zeng G-Q, Lu K-D (2019). Constrained multi-objective population extremal optimization based economic-emission dispatch incorporating renewable energy resources. Renew. Energy.

[24] Kennedy, J., Kennedy, J. F. & Eberhart, R. C. *Swarm intelligence* (Morgan Kaufmann, 2001).

[25] AlRashidi M, El-Hawary M (2007). Hybrid particle swarm optimization approach for solving the discrete opf problem considering the valve loading effects. IEEE Trans. Power Syst..

[26] Park J-B, Lee K-S, Shin J-R, Lee K-S (2005). A particle swarm optimization for economic dispatch with nonsmooth cost functions. IEEE Trans. Power Syst..

[27] Selvakumar AI, Thanushkodi K (2007). A new particle swarm optimization solution to nonconvex economic dispatch problems. IEEE Trans. Power Syst..

[28] Coello, C. A. C. *Evolutionary algorithms for solving multi-objective problems* (Springer, 2007).

[29] Santana-Quintero, L. V., Montano, A. A. & Coello, C. A. C. A review of techniques for handling expensive functions in evolutionary multi-objective optimization. In *Computational intelligence in expensive optimization problems* 29–59 (2010).

[30] Klinkenberg, J.-W., Emmerich, M. T., Deutz, A. H., Shir, O. M. & Bäck, T. A reduced-cost sms-emoa using kriging, self-adaptation, and parallelization. In *Multiple Criteria Decision Making for Sustainable Energy and Transportation Systems: Proceedings of the 19th International Conference on Multiple Criteria Decision Making, Auckland, New Zealand, 7th-12th January 2008*, 301–311 (Springer, 2010).

[31] Li B, Li J, Tang K, Yao X (2015). Many-objective evolutionary algorithms: A survey. ACM Comput. Surv. (CSUR).

[32] Cheng R, Jin Y, Olhofer M (2016). Test problems for large-scale multiobjective and many-objective optimization. IEEE Trans. Cybern..

[33] Antonio, L. M. & Coello, C. A. C. Use of cooperative coevolution for solving large scale multiobjective optimization problems. In *2013 IEEE Congress on Evolutionary Computation*, 2758–2765 (IEEE, 2013).

[34] Lopez, E. M., Antonio, L. M. & Coello Coello, C. A. A gpu-based algorithm for a faster hypervolume contribution computation. In *International Conference on Evolutionary Multi-Criterion Optimization*, 80–94 (Springer, 2015).

[35] Hernández Gómez, R., Coello Coello, C. A. & Alba, E. A parallel version of sms-emoa for many-objective optimization problems. In *International Conference on Parallel Problem Solving from Nature*, 568–577 (Springer, 2016).

[36] Glasmachers, T. Optimized approximation sets for low-dimensional benchmark pareto fronts. In *International Conference on Parallel Problem Solving from Nature*, 569–578 (Springer, 2014).

[37] Aguirre, H., Liefooghe, A., Verel, S. & Tanaka, K. A study on population size and selection lapse in many-objective optimization. In *2013 IEEE Congress on Evolutionary Computation*, 1507–1514 (IEEE, 2013).

[38] Wang L, Singh C (2007). Environmental/economic power dispatch using a fuzzified multi-objective particle swarm optimization algorithm. Electr. Power Syst. Res..

[39] Kitamura, S., Mori, K., Shindo, S., Izui, Y. & Ozaki, Y. Multiobjective energy management system using modified mopso. In *2005 IEEE International Conference on Systems, Man and Cybernetics*, vol. 4, 3497–3503 (IEEE, 2005).

[40] Hazra J, Sinha AK (2007). Congestion management using multiobjective particle swarm optimization. IEEE Trans. Power Syst..

[41] Mostaghim, S. & Teich, J. Strategies for finding good local guides in multi-objective particle swarm optimization (mopso). In *Proceedings of the 2003 IEEE Swarm Intelligence Symposium. SIS’03 (Cat. No. 03EX706)*, 26–33 (IEEE, 2003).

[42] Vlachogiannis JG, Lee KY (2005). Determining generator contributions to transmission system using parallel vector evaluated particle swarm optimization. IEEE Trans. Power Syst..

[43] Coello Coello CA (1999). A comprehensive survey of evolutionary-based multiobjective optimization techniques. Knowl. Inf. Syst..

[44] Hao H, Zhang X, Zhou A (2024). Enhancing saeas with unevaluated solutions: A case study of relation model for expensive optimization. Sci. China Inf. Sci..

[45] Wang S, Zhou A (2023). Regularity evolution for multiobjective optimization. IEEE Trans. Evol. Comput..

[46] Zeng G-Q, Xie X-Q, Chen M-R, Weng J (2019). Adaptive population extremal optimization-based pid neural network for multivariable nonlinear control systems. Swarm Evol. Comput..

[47] Jin X, He T, Lin Y (2019). Multi-objective model selection algorithm for online sequential ultimate learning machine. EURASIP J. Wirel. Commun. Netw..

[48] Deng W (2022). An enhanced fast non-dominated solution sorting genetic algorithm for multi-objective problems. Inf. Sci..

[49] Lakhan A (2022). Federated learning-aware multi-objective modeling and blockchain-enable system for iiot applications. Comput. Electr. Eng..

[50] Dashtdar M (2022). Solving the environmental/economic dispatch problem using the hybrid fa-ga multi-objective algorithm. Energy Rep..

[51] Benyekhlef, L., Lahouari, B. & Abdelkader, S. Static/dynamic economic-environmental dispatch problem using cuckoo search algorithm. *Power Electron. Green Energy Convers.* 453–473 (2022).

[52] Rizki A, Habachi R, Tahiry K, Echchatbi A (2022). Economic dispatch problem in smart grid system with considerations for pumped storage. Bull. Electr. Eng. Inf..

[53] Adnan, S., Islam, M. R., Shafiullah, M., Hoque, S. & Azam, M. S. Bald eagle search optimization algorithm for economic dispatch problem with renewable energy integration. In *2023 XIX International Scientific Technical Conference Alternating Current Electric Drives (ACED)*, 1–6 (IEEE, 2023).

[54] Younes, M., Khodja, F. & Kherfene, R. L. Economic and emission dispatch problems using a new hybrid algorithm. In *the 2013 International Conference on Environment, Energy, Ecosystems and Development*, 119–126 (2013).

[55] Acharya, S., Sivarajan, G., Kumar, D. V. & Srikrishna, S. Modeling combined economic emission dispatch for renewable energy system via levy-based glowworm swarm optimization. *Kybernetes* (2022).

[56] Sakthivel V, Sathya P (2022). Multi-area economic environmental dispatch using multi-objective squirrel search algorithm. Evol. Syst..

[57] Rawa M (2021). Economical-technical-environmental operation of power networks with wind-solar-hydropower generation using analytic hierarchy process and improved grey wolf algorithm. Ain Shams Eng. J..

[58] Iqbal MN (2022). Solution of economic dispatch problem using hybrid multi-verse optimizer. Electr. Power Syst. Res..

[59] Abido M (2002). Optimal design of power-system stabilizers using particle swarm optimization. IEEE Trans. Energy Convers..

[60] Khalil, M. I. K., Rahman, I. U., Zakarya, M. & Khan, M. A neighborhood-aware multi-markovian switching particle swarm optimization technique for solving complex and expensive problems. *Soft Computing* (2023).

[61] Zhan Z-H, Zhang J, Li Y, Chung H-H (2009). Adaptive particle swarm optimization. IEEE Trans. Syst. Man Cybern. Part B: Cybern..

[62] Tang Y, Wang Z, Fang J-A (2011). Parameters identification of unknown delayed genetic regulatory networks by a switching particle swarm optimization algorithm. Expert Syst. Appl..

[63] Rahman, I. U. *Novel Particle Swarm Optimization Algorithms with Applications in Power Systems*. Ph.D. thesis, Brunel University London (2016).

[64] Rahman IU (2020). An n-state Markovian jumping particle swarm optimization algorithm. IEEE Trans. Syst. Man Cybern.: Syst..

[65] Rahman IU, Zakarya M, Raza M, Khan R (2020). An n-state switching pso algorithm for scalable optimization. Soft. Comput..

[66] Ababneh JI, Bataineh MH (2008). Linear phase fir filter design using particle swarm optimization and genetic algorithms. Digit. Signal Process..

